# Axial Skeletal Assessment in Osteoporosis Using Radiofrequency Echographic Multi-spectrometry: Diagnostic Performance, Clinical Utility, and Future Directions

**DOI:** 10.7759/cureus.86625

**Published:** 2025-06-23

**Authors:** Mohammed As'ad

**Affiliations:** 1 Corporate Quality &amp; Patient Safety Department, Dr. Sulaiman Al Habib Medical Group, Riyadh, SAU

**Keywords:** bone mineral density, diagnostic ultrasound, fracture risk prediction, osteoporosis diagnosis, radiofrequency echographic multispectrometry, rems, skeletal fragility, spectral ultrasound analysis, trabecular bone score, ultrasound bone assessment

## Abstract

Osteoporosis, a prevalent skeletal disorder, necessitates accurate and accessible diagnostic tools for effective disease management and fracture prevention. While dual-energy X-ray absorptiometry (DXA) remains the clinical standard for bone mineral density (BMD) assessment, its limitations, including ionizing radiation exposure and susceptibility to artifacts, underscore the need for alternative technologies. Ultrasound-based methods have emerged as promising radiation-free alternatives, with radiofrequency echographic multi-spectrometry (REMS) representing a significant advancement in axial skeleton assessment, specifically at the lumbar spine and proximal femur. REMS analyzes unfiltered radiofrequency ultrasound signals, providing not only BMD estimates but also a novel fragility score (FS), which reflects bone quality and microarchitectural integrity. This review critically evaluates the underlying principles, diagnostic performance, and clinical applications of REMS. It compares REMS with DXA, quantitative computed tomography (QCT), and trabecular bone score (TBS), highlighting REMS’s potential advantages in artifact-prone scenarios and specific populations, including children and patients with secondary osteoporosis. The clinical utility of REMS in fracture risk prediction and therapy monitoring is explored alongside its operational precision, cost-effectiveness, and portability. In addition, the integration of artificial intelligence (AI) within REMS software has enhanced its capacity for artifact exclusion and automated spectral interpretation, improving usability and reproducibility. Current limitations, such as the need for broader validation and guideline inclusion, are identified, and future research directions are proposed. These include multicenter validation studies, development of pediatric and secondary osteoporosis reference models, and deeper evaluation of AI-driven enhancements. REMS offers a compelling, non-ionizing alternative for axial bone health assessment and may significantly advance the diagnostic landscape for osteoporosis care.

## Introduction and background

Osteoporosis is a systemic skeletal disorder marked by diminished bone mass and disrupted microarchitecture, culminating in greater bone fragility and elevated fracture risk [1]. Often dubbed a "silent disease," it progresses unnoticed until a fracture occurs, imposing serious health burdens and economic strain globally [1]. One in three women and one in five men over 50 are projected to sustain an osteoporotic fracture, commonly affecting the hip and spine [2]. These events frequently result in chronic pain, functional decline, and increased mortality, underscoring the urgency for accurate diagnostics and proactive interventions. Bone mineral density (BMD) assessment remains central to diagnosing osteoporosis and estimating fracture risk. Dual-energy X-ray absorptiometry (DXA), FDA-approved since 1988, remains the gold standard, particularly for the lumbar spine and proximal femur [3]. While offering areal BMD (aBMD) values using low-dose X-rays, DXA is constrained by its two-dimensional imaging and susceptibility to artifacts, including osteophytes, vascular calcifications, and vertebral deformities [4]. It also offers minimal insight into bone microarchitecture or material strength, both crucial to bone resilience [5]. Quantitative computed tomography (QCT), in contrast, provides volumetric BMD (vBMD) and distinguishes trabecular from cortical bone, enabling more precise structural assessment [4]. However, its elevated radiation dose, higher cost, and limited availability reduce its routine clinical appeal [3]. Quantitative ultrasound (QUS), often applied at peripheral sites like the heel or radius, offers radiation-free, portable, and affordable screening, but its predictive accuracy for central fractures and correlation with axial DXA measurements remains limited [5].

Despite their widespread use, current densitometry tools present significant clinical gaps. The reliance on ionizing radiation in DXA and QCT restricts their use in sensitive populations and limits monitoring frequency [4]. Accessibility and cost barriers persist, especially in low-resource settings [3]. DXA’s vulnerability to anatomical artifacts can misrepresent BMD, potentially leading to incorrect clinical decisions [6]. More critically, BMD accounts for only 50-70% of bone strength variability, leaving substantial aspects of bone quality, such as microarchitecture, turnover, and material composition, unmeasured [6]. This inadequacy drives the demand for diagnostic technologies offering a more holistic view of bone health [5]. Recent advances have shifted attention toward ultrasound-based solutions targeting the axial skeleton. While peripheral QUS has proven useful for preliminary risk stratification, efforts to reliably assess axial sites using ultrasound have remained limited. Radiofrequency echographic multi-spectrometry (REMS) introduces a novel approach by capturing unfiltered radiofrequency (RF) signals reflected from bone tissue at the lumbar spine and proximal femur [3,7]. Unlike conventional ultrasound, REMS analyzes these raw signals to extract detailed spectral features, yielding not only BMD-equivalent measurements but also a fragility score (FS) indicative of bone quality [3]. REMS offers a non-ionizing, artifact-resistant, and potentially more accessible alternative to traditional methods. Its clinical adoption reflects a growing recognition of the limitations of current techniques and the promise of ultrasound innovations in addressing longstanding diagnostic deficiencies in osteoporosis care.

## Review

Principles of ultrasound in bone assessment

Ultrasound imaging utilizes high-frequency sound waves generated by piezoelectric transducers to visualize biological tissues. The propagation speed of these waves is dependent on the density and stiffness of the medium, with the relationship between velocity (c), frequency (f), and wavelength (λ) given by the equation c=fλ [8]. As these waves travel through the body, they experience attenuation, a progressive energy loss that limits penetration depth but is crucial for image contrast. This loss occurs through absorption, reflection, and scattering, and it is more pronounced at higher frequencies, which otherwise offer better image resolution [9]. Reflections, or echoes, are generated at the boundaries between tissues with different acoustic impedances, a characteristic defined as the product of tissue density and acoustic velocity (Z=ρc), and are then detected to form an image [8,9]. Bone tissue presents a distinct acoustic profile because its high density and acoustic velocity result in a significantly greater acoustic impedance than that of soft tissue [8]. Consequently, a substantial portion of the ultrasound wave is reflected at the soft tissue-bone interface, causing bone to appear as a bright, hyperechoic structure [9]. While this strong reflection was historically seen as a limitation, the backscattered waves contain valuable data. The porous, lattice-like structure of trabecular bone, in particular, scatters ultrasound waves in multiple directions. The characteristics of this scattering are directly influenced by key components of bone microarchitecture, such as the density, spacing, and connectivity of the trabeculae [6].

QUS emerged as a non-ionizing, portable method for assessing bone, primarily at peripheral sites like the calcaneus, by measuring parameters such as broadband ultrasound attenuation (BUA) and speed of sound (SOS) [5]. Although early QUS devices showed a moderate ability to predict fracture risk, their utility for diagnosing osteoporosis by WHO standards or monitoring treatment was limited. A significant drawback was the difficulty in adapting these techniques for accurate assessment of the clinically critical axial sites, namely the lumbar spine and hip [4,5]. These challenges paved the way for new-generation technologies like REMS, which represents a significant evolution. By analyzing the raw RF backscatter signals from axial bones, REMS aims to overcome the historical limitations and provide a more direct and comprehensive assessment of bone health [4].

REMS Technology

REMS is an innovative ultrasound-based technology designed for the non-ionizing assessment of BMD and bone quality at axial skeletal sites, primarily the lumbar spine and proximal femur [4]. It distinguishes itself from conventional ultrasound and earlier QUS methods by its unique approach to signal acquisition and analysis.

Fundamental Principles and Acquisition Protocol

REMS technology analyzes native, raw, unfiltered RF signals, preserving the complete waveform and its spectral information on bone microarchitecture - data typically lost during conventional B-mode image reconstruction [7]. The acquisition protocol uses a standard 3.5 MHz convex probe for the lumbar spine (L1-L4) and proximal femur (femoral neck, total hip) [3]. The process requires a supine patient, with demographic data (age, sex, height, weight, ethnicity) entered for reference database matching [1]. Guided by B-mode imaging, the operator places the probe on the abdomen or upper thigh, manually adjusting acquisition parameters like scan depth and transducer focus to ensure optimal positioning of the bone interface [1,4]. The software-assisted scan acquires thousands of RF A-lines in approximately 80 seconds for the lumbar spine and 40 seconds for the femoral neck [10]. Simultaneously, the software uses B-mode images to automatically detect bone interfaces and define regions of interest (ROIs) for analysis [3].

**Figure 1:**
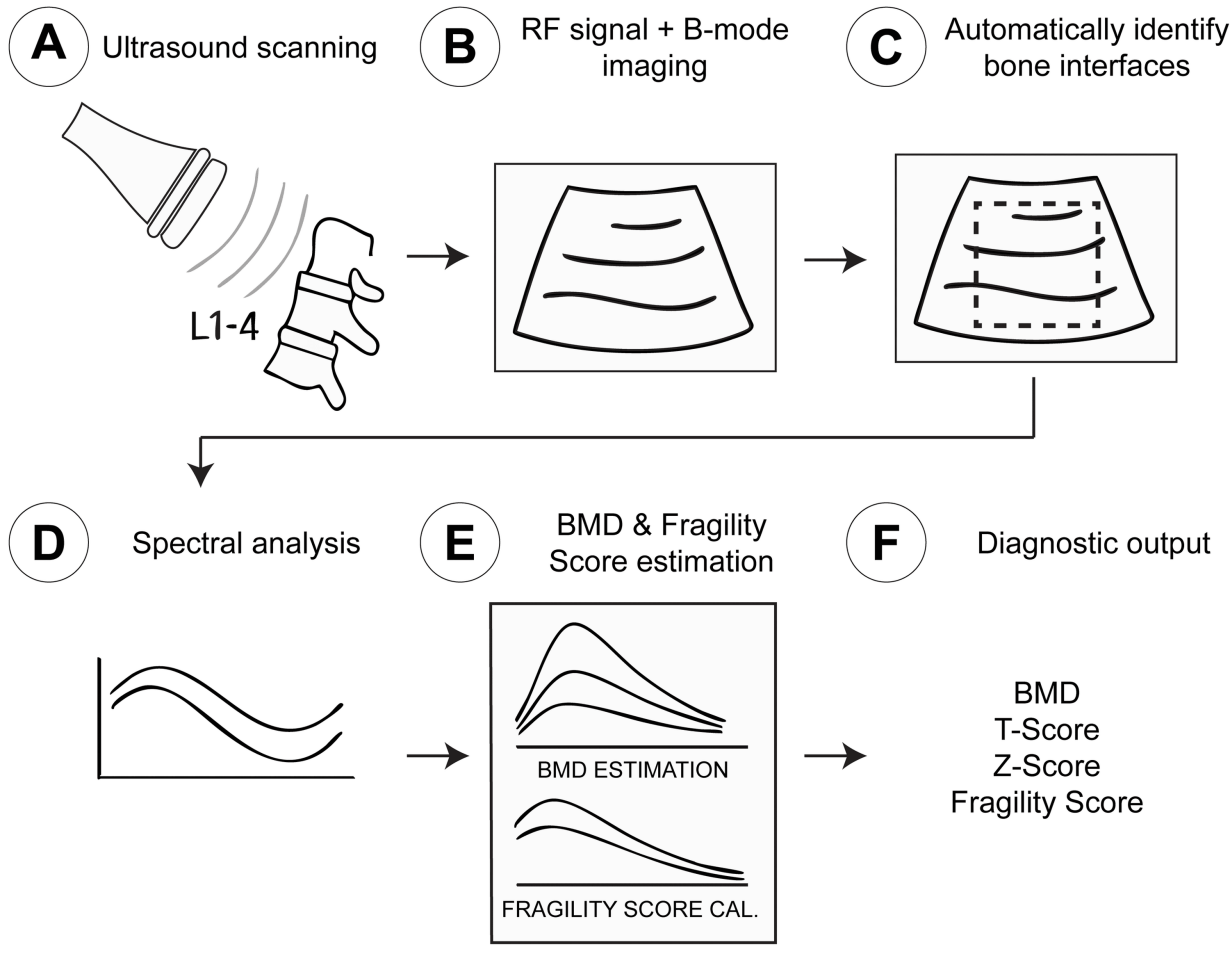
Conceptual overview of REMS (A) An ultrasound probe (typically 3.5 MHz convex array) is used to scan axial skeletal sites, such as the lumbar spine (L1–L4) or proximal femur. (B) The probe emits ultrasound waves, and the backscattered raw RF signals are acquired. Simultaneously, B-mode images are generated for anatomical localization and operator guidance. (C) The REMS software automatically identifies bone interfaces within the B-mode image and defines ROIs. (D) The corresponding raw RF signals from these ROIs undergo spectral analysis. A key step involves the automatic identification and exclusion of signals deemed to be artifacts (e.g., from osteophytes or vascular calcifications) based on their atypical spectral characteristics. (E) The processed patient-specific RF spectra are then compared against extensive, proprietary reference spectral models stratified by age, sex, BMI, and anatomical site. (F) The final diagnostic output includes BMD (in g/cm²), T-score, Z-score, and an independent fragility score (FS, 0-100), reflecting bone quality and microarchitectural integrity. REMS, radiofrequency echographic multi-spectrometry; RF, radiofrequency; ROIs, regions of interest; BMD, bone mineral density. This figure was created by the author based on the technical descriptions in Adami G, et al. [11].

A key feature is the automatic exclusion of artifacts by analyzing the spectral characteristics of acquired RF signals. Atypical signals from osteophytes, dense vascular calcifications, or metallic implants are recognized as non-representative and discarded, which enhances accuracy where DXA measurements might be confounded [1]. The integration of B-mode for anatomical localization and ROI definition with diagnostic analysis performed on the corresponding raw RF data is a distinctive REMS feature (Figure 1) [3].

Data Processing: Osteoporosis Score and FS

Following RF signal acquisition and ROI identification, REMS software compares the patient’s RF frequency spectra against a large database of reference models. This database contains spectral "fingerprints" from individuals with known bone status and fracture histories, matched for demographic variables including gender, age, and BMI [1]. From this, an Osteoporosis Score (OS) is derived, quantifying the spectral similarity to osteoporotic models and often corresponding to the percentage of analyzed ROI segments classified as "osteoporotic" [1]. The OS is a REMS-specific internal metric that is not standardized in clinical practice but is used within the REMS system to compute conventional diagnostic outputs such as BMD, T-score, and Z-score. The OS is then converted into BMD (g/cm²), T-score, and Z-score using site-specific linear equations and comparison with standard reference populations [1].

A distinctive output is the FS, a dimensionless 0-100 parameter designed to assess bone quality and structural integrity independent of BMD, with higher scores suggesting increased fragility [3]. The FS is calculated by comparing patient spectra against different reference models derived from cohorts with and without documented fragility fractures, aiming to capture aspects of bone microarchitecture not reflected in BMD alone [3]. This is based on the premise that fragile bone exhibits different ultrasound backscatter characteristics due to microstructural alterations [3]. REMS reports often provide a scattergram plotting the patient's FS against age with color-coded risk zones derived from the reference database's fracture history [6].

The dual output of BMD-equivalent parameters and an independent FS is a significant departure from traditional densitometry. This multi-parametric assessment holds potential for a more nuanced evaluation of fracture risk, with the OS bridging to familiar diagnostics and the FS introducing a new dimension of bone's intrinsic fracture resistance. The automatic exclusion of artifactual signals during the spectral analysis is a critical element that improves the reliability of both OS and FS calculations.

Diagnostic performance of REMS

The clinical acceptance of any new diagnostic technology hinges on its performance characteristics, particularly its accuracy, precision, and ability to correctly classify disease states compared to established standards. REMS technology has been subjected to numerous studies evaluating these aspects, primarily against DXA, but also with considerations for QCT and TBS.

Comparison With DXA (Accuracy, Sensitivity, Specificity, and Artifacts)

A substantial body of evidence compares REMS with DXA, the clinical gold standard. Multiple studies show a strong, significant correlation between REMS and DXA for BMD and T-scores at the lumbar spine and femoral neck, with Pearson correlation coefficients (r) frequently above 0.90 [1,12]. Diagnostic agreement is good to excellent (Cohen's kappa, k ≈ 0.8), indicating substantial concordance in classifying patients into normal, osteopenic, or osteoporotic categories per WHO criteria across various populations [1]. REMS demonstrates high sensitivity and specificity for differentiating osteoporotic from non-osteoporotic individuals versus DXA; reported values often exceed 90-92% for both sites [1]. For instance, a European multicenter study reported sensitivities of 90.9% (lumbar spine) and 90.4% (femoral neck) and specificities of 95.1% and 95.5%, respectively [12].

A highlighted advantage is REMS's potential for more accurate BMD assessment where DXA is compromised by artifacts like degenerative changes (osteoarthritis, spondyloarthrosis), vertebral compression fractures, or extraskeletal calcifications (e.g., aortic), which can artificially inflate DXA values [1]. REMS technology is designed to identify and exclude these artifactual signals via spectral analysis of raw RF signals. Consequently, in patients with spinal osteoarthritis, REMS-derived T-scores are often significantly lower than DXA T-scores [12]; one study by Betancur et al. [6] found that REMS classified 35.1% of women with spondyloarthrosis as osteoporotic compared to 9.3% by DXA. Furthermore, they pointed out that REMS has a utility in assessing sites with metallic implants where DXA cannot be reliably used.

Furthermore, REMS has exhibited excellent intra- and inter-operator precision, with a root-mean-square coefficient of variation (RMS-CV) typically below 0.5% (e.g., 0.38% spine, 0.32% femur), which is superior to DXA's precision (often 1-2%) [3,13]. This results in a smaller least significant change (LSC) for REMS (e.g., ~1% spine, 0.88% femur) compared to DXA (typically 5-7%), suggesting REMS can detect smaller BMD changes over shorter intervals [4]. These findings support the diagnostic equivalence of REMS to DXA, with potential superiority in common clinical situations plagued by artifacts, which could refine diagnostic accuracy and clinical management.

Comparison With QCT and TBS

Direct diagnostic accuracy studies comparing REMS with QCT are less extensively reported than those with DXA. Both techniques offer insights into trabecular bone; QCT provides true vBMD and can separately analyze trabecular and cortical compartments, avoiding superficial artifacts, while REMS analyzes backscattered RF signals presumed to originate from the trabecular layer [4,6]. Key differences are that QCT involves ionizing radiation, is more expensive, and is less portable than the radiation-free REMS, limiting QCT's routine use for screening despite its value as a research tool [3]. Future studies directly comparing the REMS FS with QCT-derived parameters are warranted.

REMS is also compared with the trabecular bone score (TBS), a texture analysis of lumbar spine DXA images that provides an indirect measure of trabecular microarchitecture independent of BMD [12]. As the REMS FS also aims to assess bone quality, this comparison is relevant. Studies report that REMS performs similarly to DXA in the context of TBS-assessed integrity, and the correlation between BMD (from either REMS or DXA) and TBS is, as expected, low to moderate (e.g., r=0.27 for REMS-BMD vs. TBS; r=0.39 for DXA-BMD vs. TBS) [7]. This suggests that BMD (mineral quantity) and TBS (structural organization) provide complementary information. The utility of REMS is further supported by the similar performance of its T-scores within FRAX and NOGG fracture risk frameworks [7]. Because the REMS FS derives from direct spectral analysis rather than DXA image texture, more comparisons of its fracture prediction ability versus TBS are needed.

While REMS-derived BMD shows strong concordance with DXA, its unique contribution, like that of QCT and TBS, may be its ability to provide information beyond DXA alone, especially regarding artifact management and bone quality [14]. The low correlation between any source of BMD and TBS underscores that these are not redundant measures. The REMS FS, by its design, aims to be a more direct measure of this qualitative aspect of bone strength than BMD [6]. A comparative overview of REMS, DXA, QCT, and TBS is presented in Table 1.

**Table 1:** Comparative overview of axial bone assessment technologies Note: This table was created by the author to provide a comparative overview, based on information presented in the systematic review by Icătoiu E, et al. [3]. Costs and scan times are indicative and can vary. DXA, dual-energy X-ray absorptiometry; REMS, radiofrequency echographic multi-spectrometry; QCT, quantitative computed tomography; TBS, trabecular bone score; aBMD, areal bone mineral density; US-BMD, ultrasound-derived bone mineral density; vBMD, volumetric bone mineral density; RF, radiofrequency; OA, osteoarthritis; LSC, least significant change; FS, fragility score; VFA, vertebral fracture assessment.

Feature	DXA	REMS	QCT	TBS
Principle	X-ray attenuation	Ultrasound RF spectral analysis	X-ray attenuation (CT-based)	DXA image texture analysis
Radiation dose	Low	None	Moderate to high	Same as DXA (derived from DXA scan)
Portability	Limited (fixed units)	High (portable devices)	Very limited (fixed CT scanners)	N/A (software for DXA)
Axial sites measured	Lumbar spine, proximal femur, forearm	Lumbar spine, proximal femur	Lumbar spine, proximal femur	Lumbar spine (from DXA image)
Key parameters	aBMD, T-score, Z-score	US-BMD, T-score, Z-score, fragility score (FS)	vBMD, T-score (derived)	TBS value, TBS Z-score
Susceptibility to OA artifacts	High (can falsely elevate BMD)	Low (designed to exclude artifacts)	Moderate (can isolate trabecular bone)	Low to moderate (less affected than BMD)
Susceptibility to metallic artifacts	High	Moderate (can assess if enough bone available)	Very high	Same as DXA
Assessment of bone quality/microarchitecture	Indirect (limited), VFA capability	Direct (FS), some microarchitectural insights from RF analysis	Direct (trabecular/cortical separation, some microarchitectural parameters with HR-pQCT)	Indirect (texture analysis)
Typical scan time	5-15 minutes	~2 minutes (spine + femur combined: ~80s + ~40s)	10-20 minutes	N/A (post-processing of DXA scan)
Cost (general indication)	Moderate	Potentially lower to moderate	High	Additional software cost for DXA
Precision (typical LSC)	~5-7%	~1-1.5%	Variable, generally good	~1.5-3% (site dependent)

Clinical applications of REMS

The clinical utility of REMS extends beyond simple diagnosis of osteoporosis, encompassing fracture risk prediction, potential for monitoring therapy, and application in various special populations where traditional methods may be challenging. A summary of the key clinical studies supporting these applications is presented in Table 2.

**Table 2:** Summary of key clinical studies on REMS performance LS, lumbar spine; FN, femoral neck; AUC, area under the curve; FS, fragility score; AUC, area under the curve; REMS, radiofrequency echographic multi-spectrometry; DXA, dual-energy X-ray absorptiometry; BMD, bone mineral density; ICER, incremental cost-effectiveness ratio; QALY, quality-adjusted life year; RMS-CV, root-mean-square coefficient of variation; T2DM, type 2 diabetes mellitus.

Reference	Study Design	Population	Key Comparators	Primary Outcomes Assessed	Key Findings for REMS
Vieira & Santos, 2025 [1]; Sözen et al., 2017 [2]	Prospective observational cohort (up to 5-year follow-up)	>1500 women and men (30-90 years)	DXA T-score, REMS T-score	Incident fragility fracture prediction (generic osteoporotic and hip) by FS	FS showed significantly higher AUCs for fracture prediction (e.g., generic osteoporotic: FS AUC 0.811 women, 0.780 men) compared to REMS or DXA T-scores.
Zambito et al., 2025 [4]	Multicenter cross-sectional	~1600-2700 postmenopausal women	DXA	Diagnostic accuracy (BMD, T-score), concordance, sensitivity, specificity, precision	Strong correlation with DXA (r>0.9); high sensitivity (>90%) and specificity (>90-95%); good concordance (kappa ~0.8); high precision (RMS-CV <0.5%)
Betancur et al., 2025 [6]	Multicenter cross-sectional	603 Caucasian males (30-90 years)	DXA	Diagnostic accuracy (BMD, T-score), concordance, sensitivity, specificity, precision in men	Strong correlation with DXA T-scores (LS r=0.91, FN r=0.90); high sensitivity (LS 90.1%, FN 90.9%) and specificity (LS 93.6%, FN 94.6%); good precision.
Diez-Perez et al., 2019 [7]	Economic modeling study	US women aged ≥ 50 years with osteoporosis	No diagnosis/treatment	Cost-effectiveness (ICER per QALY), fractures prevented, QALYs saved	REMS + treatment is cost-effective ($33,891-$49,198/QALY). 5% increased diagnosis could prevent 100,000 fractures.
Diez-Perez et al., 2019 [7]	Cost-minimization analysis	Italian National Health Service perspective	DXA	Direct healthcare costs	REMS associated with mean saving of €40.0 per patient compared to DXA.
Di Paola et al., 2019 [10]	Precision study	Patients undergoing REMS	Intra-operator and inter-operator variability	Precision (RMS-CV), repeatability (LSC)	Excellent intra-operator precision (RMS-CV LS: 0.47%, FN: 0.32%) and inter-operator repeatability (RMS-CV LS: 0.55%, FN: 0.51%).
Pisani et al., 2023 [15]	Cross-sectional/comparative	Women with spinal osteoarthritis; women with T2DM	DXA	Diagnostic accuracy in the presence of OA; bone status assessment in T2DM	REMS T-scores lower than DXA in OA, classifying more as osteoporotic (e.g., 35.1% vs 9.3% with OA); REMS potentially more sensitive in T2DM.

Fracture Risk Prediction

An accurate prediction of future fragility fractures is a primary goal of bone assessment. Longitudinal studies indicate that T-scores derived from REMS are effective in predicting incident fragility fractures, with performance generally comparable to DXA-derived T-scores [11,15], thus establishing REMS-BMD as a valid parameter for risk stratification [16]. The FS, designed to be independent of BMD by reflecting bone quality and microarchitectural integrity, is emerging as a potentially more powerful predictor [7]. Cross-sectional studies show that FS values are significantly higher (worse) in patients with prevalent fragility fractures compared to non-fractured individuals [3]. More importantly, a key five-year prospective study demonstrated that the FS had a superior ability to predict incident osteoporotic fractures compared to T-scores from either REMS or DXA [17]. In that study, the area under the ROC curve (AUC) for FS in predicting generic osteoporotic fractures was 0.811 for women and 0.780 for men; for hip fractures, AUCs were 0.780 for women and 0.809 for men, values consistently and significantly higher than T-score AUCs (which ranged from 0.472 to 0.709) and which maintained superiority after age and BMI adjustment [15,17]. Good correlation has also been reported between the REMS FS and 10-year fracture probability calculated by FRAX® [3]. Furthermore, REMS technology allows for a combined risk assessment, often presented in a matrix format, that uses both T-score and FS to provide a categorized five-year fracture risk estimate (e.g., R1-R7 scale) [6]. The ability of FS to capture non-BMD determinants of bone strength is the basis for its enhanced predictive capability and could significantly refine risk assessment.

Monitoring Osteoporosis Therapy

REMS offers potential advantages for monitoring osteoporosis therapy due to its radiation-free nature, which allows for more frequent assessments, and its high precision (low LSC), which may detect treatment-induced changes earlier than DXA. While large-scale comparative trials are evolving, preliminary evidence exists. The ISCD has weakly favored REMS for short-term monitoring, and studies have demonstrated its feasibility for six-month follow-up intervals, such as in breast cancer patients on aromatase inhibitors or following denosumab administration, which contrasts with DXA’s typical 1-2-year intervals [14]. Some sources claim REMS is significantly more sensitive than DXA for detecting small changes (e.g., "more sensitive for the smallest detectable difference"), though such claims require more robust substantiation [3]. However, the smaller LSC of REMS (~1-1.5%) compared to DXA (~5-7%) inherently supports its potential for detecting smaller changes [16]. Specific evidence shows REMS has been used to track changes with denosumab [14]. Specific studies on REMS performance for monitoring bisphosphonates and teriparatide are less prominent [12]. The theoretical advantages are strong, but more extensive clinical trial data are needed to establish REMS's role in therapy monitoring.

Use in Special Populations

REMS technology is promising for populations where DXA has limitations. In men, REMS provides BMD and T-scores that correlate highly with DXA, with good diagnostic accuracy, and the FS has shown predictive value for fractures [1,15]. For pediatrics and adolescents, its radiation-free nature is a major advantage, and its spectral analysis may be less confounded by bone size than DXA's areal BMD [1,18,19]. Research is underway to validate pediatric algorithms, including fetal bone densitometry, but the development of specific reference databases is crucial [18]. In pregnancy and lactation, REMS offers a safe alternative to the contraindicated DXA [1].

REMS is also valuable in secondary osteoporosis due to its artifact resistance. This includes potential use in glucocorticoid-induced osteoporosis (GIO) [20] and demonstrated effectiveness in rheumatoid arthritis (RA) [1]. In organ transplant recipients, particularly kidney transplant patients, where vascular calcifications can limit DXA, REMS has shown promise by avoiding these artifacts [4]; data on hematopoietic stem cell transplant recipients is less direct [21]. Finally, in type 2 diabetes mellitus (T2DM), where fracture risk is elevated despite normal or high DXA-BMD, REMS may be more sensitive, with some studies showing that it classifies a higher percentage of T2DM patients as osteoporotic than DXA [4]. The versatility of REMS positions it as a valuable tool for these and other clinical scenarios that challenge traditional densitometry.

Cost-effectiveness and usability of REMS

Beyond diagnostic performance, the practical aspects of a technology, including its economic viability and ease of use, are critical for widespread clinical adoption. REMS technology has been evaluated for both its cost-effectiveness and its operational characteristics.

Economic Evaluations

Beyond diagnostic performance, economic viability is critical for clinical adoption. In the US context, an economic modeling study of women aged 50 and older found REMS to be a cost-effective strategy for osteoporosis diagnosis and treatment [22]. The incremental cost-effectiveness ratio for REMS was estimated at $33,891 per quality-adjusted life year (QALY) gained with full medication adherence, and $49,198 per QALY under real-world adherence, both well below the common US threshold of $100,000 per QALY. The model projected that a modest 5% increase in diagnosis and treatment using REMS could prevent over 100,000 fractures and save approximately 43,500 QALYs over a lifetime, reducing fracture-related costs [22]. From the Italian National Health Service (NHS) perspective, a cost-minimization analysis indicated that REMS is associated with lower direct healthcare costs than DXA, with a mean saving of €40.0 per patient; specific costs for professionals (€31.9 vs. €48.8) and examinations/tests (€45.1 vs. €68.2) were lower for REMS versus DXA, respectively [16]. There are also suggestions that the REMS device may be less expensive than a DXA densitometer [7]. These evaluations suggest that REMS may present economic benefits by improving diagnostic access and reducing long-term fracture costs.

Learning Curve, Portability, and Accessibility

A significant practical advantage of REMS devices is their portability, unlike fixed DXA machines. REMS systems can be transported to various settings, including primary care offices, community clinics, and a patient's bedside, greatly enhancing accessibility for frail, immobilized, or remote populations and for opportunistic screening [1]. Its radiation-free nature also obviates the need for dedicated shielded rooms. REMS is often described as simpler to perform than DXA, with software that automates bone interface detection, ROI placement, and artifact exclusion, and with short scan times of approximately 1-2 minutes per site [3,7]. However, while automated, operator proficiency remains important for reliable results. Initial training on anatomy, patient positioning, probe handling, and setting scan parameters is necessary, and adherence to standardized procedures is crucial for reproducibility [4]. A learning curve exists, as some studies note scan exclusions due to operator error or difficulty identifying bone structures in early experiences with the technology [23]. Therefore, the development of comprehensive training and quality assurance programs is important for successful widespread implementation. The combination of favorable economics, portability, and user-friendly design (with appropriate training) positions REMS to broaden osteoporosis screening, though ensuring consistent operator competency is paramount.

Role of Artificial Intelligence in REMS and Future Ultrasound Bone Densitometry

Artificial intelligence (AI) is fundamentally integrated into REMS technology, underpinning its core operational principles rather than serving as a mere add-on. The system's "intelligence" is embedded in sophisticated algorithms that automatically analyze B-mode ultrasound images to identify bone interfaces and define ROIs, thereby reducing operator variability in ROI placement [1]. A critical AI-driven function is the spectral analysis of raw RF signals, where the system is trained to recognize bone-specific spectral signatures and automatically exclude anomalous signals from artifacts like osteophytes or calcifications [1]. Furthermore, the calculation of the OS and FS heavily relies on AI-powered pattern recognition, comparing patient RF spectra against extensive proprietary databases of reference spectral models matched for demographics and clinical status to classify bone segments and compute these diagnostic scores. The future integration of more advanced AI and machine learning could further refine REMS by enabling more detailed microarchitecture analysis beyond the current FS [3], improving workflow efficiency with real-time operator feedback and quality control, developing more sophisticated personalized fracture risk models, and enhancing artifact handling capabilities.

The inherent portability of REMS, combined with ongoing advancements in AI and ultrasound miniaturization, points toward a future of increasingly decentralized bone health assessment. This trend could expand point-of-care applications into primary care, emergency departments, and specialist clinics lacking dedicated imaging suites. Moreover, the development of user-friendly, AI-assisted handheld ultrasound devices raises the prospect of home-based bone health monitoring [14]. While achieving reliable axial assessment at home presents challenges [10], the potential for longitudinal data collection and patient empowerment is significant [24]. This synergy between advanced ultrasound signal processing, intrinsic AI-driven analysis in REMS, and the evolution of AI in medical imaging suggests a shift toward more precise, personalized, and accessible osteoporosis management, potentially transforming it into a more proactive and preventative model.

Limitations of REMS technology and current ultrasound methods

Despite its advancements, REMS technology and ultrasound-based bone assessment face certain limitations. Operator skill and experience remain influential factors, necessitating proper training and adherence to standardized protocols to ensure data quality and minimize variability, as a learning curve exists [5]. Extreme body habitus, particularly high BMI, can affect ultrasound signal penetration and clarity, potentially impacting measurements despite BMI-based reference database matching. REMS primarily offers densitometric and bone quality data and currently lacks the high-resolution anatomical detail required for vertebral fracture assessment, which may necessitate separate imaging [5]. Further validation across diverse ethnic populations and a broader range of specific secondary osteoporosis conditions is needed to ensure the generalizability of reference databases and confirm diagnostic accuracy [1]. While REMS T-scores correlate well with DXA, they are not always directly interchangeable, which can cause clinical confusion if not properly contextualized. The novel FS requires continued extensive longitudinal validation in diverse, independent cohorts, and the proprietary nature of its underlying algorithms can be perceived as a "black box." The technology's incorporation into widespread clinical guidelines and its general availability are still developing compared to DXA. Finally, inherent ultrasound limitations, such as potential artifacts from bowel gas, can still occasionally affect image quality, although REMS is designed to mitigate some of these.

## Conclusions

Ultrasound-based axial bone assessment, particularly through REMS, represents a significant advance in osteoporosis management. This technology offers a compelling, radiation-free alternative to traditional methods, addressing key limitations like artifact susceptibility. REMS stands as a noteworthy innovation, providing diagnostic accuracy comparable to DXA for the lumbar spine and femur, with potential superiority in patients with degenerative joint disease or other common artifacts. The novel FS, an independent measure of bone quality, enhances fracture risk prediction beyond bone density alone. The technology’s high precision, portability, and potential cost-effectiveness further support its role in improving accessibility to bone health diagnostics. By enabling earlier diagnosis and refining risk stratification, REMS is poised to make a substantial contribution to reducing the global burden of osteoporosis and improving patient care.
